# *Neoehrlichia mikurensis* in Danish immunocompromised patients: a retrospective cohort study

**DOI:** 10.1186/s12941-023-00571-5

**Published:** 2023-03-20

**Authors:** Rosa Maja Møhring Gynthersen, Mette Frimodt Hansen, Lukas Frans Ocias, Andreas Kjaer, Randi Føns Petersen, Sisse Rye Ostrowski, Lene Harritshøj, Søren Jacobsen, Ulrik Overgaard, Karen Angeliki Krogfelt, Anne-Mette Lebech, Helene Mens

**Affiliations:** 1 Department of Infectious Diseases, Copenhagen University Hospital, Rigshospitalet, Blegdamsvej 9, 2100 Copenhagen, Denmark; 2 Department of Science and Environment, Roskilde University, Universitetsvej 1, 4000 Roskilde, Denmark; 3 Department of Clinical Microbiology, Karlstad Hospital, Region Värmland, Karlstad, Sweden; 4 Department of Clinical Physiology and Nuclear Medicine & Cluster for Molecular Imaging, Copenhagen University Hospital – Rigshospitalet & Department of Biomedical Sciences, University of Copenhagen, Copenhagen, Denmark; 5 Department of Bacteria, Fungi and Parasites, Statens Serum Institut, Copenhagen, Denmark; 6 Department of Clinical Immunology, Copenhagen University Hospital, Rigshospitalet, Copenhagen, Denmark; 7 Department of Clinical Medicine, University of Copenhagen, Copenhagen, Denmark; 8 Copenhagen Research Center for Autoimmune Connective Tissue Diseases - COPEACT, Copenhagen University Hospital, Rigshospitalet, Copenhagen, Denmark; 9 Department of Hematology, Copenhagen University Hospital, Rigshospitalet, Copenhagen, Denmark

**Keywords:** *Neoehrlichia mikurensis*, Neoehrlichiosis, Biological treatment, Tick-borne disease, B-cell depleting therapy, Immunocompromised patients

## Abstract

**Background:**

The tick-borne bacterium, *Neoehrlichia mikurensis (N. mikurensis)* can cause severe febrile illness and thromboembolic complications in immunocompromised individuals. We investigated the presence of *N. mikurensis* DNA in retrospectively collected plasma from a well-characterized cohort of Danish immunocompromised patients.

**Methods:**

Plasma samples from 239 patients with immune dysfunction related to hematological or rheumatological disease or due to immunosuppressive therapy, were retrieved from a transdisciplinary biobank (PERSIMUNE) at Rigshospitalet, Copenhagen, Denmark. Serving as immunocompetent controls, plasma samples from 192 blood donors were included. All samples were collected between 2015 and 2019. Real-time PCR targeting the *groEL* gene was used to detect *N. mikurensis* DNA. Sequencing was used for confirmation. *Borrelia burgdorferi* sensu lato IgG antibodies were detected by ELISA as a proxy of tick exposure. Prevalence was compared using Fisher’s exact test.

**Results:**

*Neoehrlichia mikurensis* DNA was detected in 3/239 (1.3%, 95% confidence interval (CI): 0.3 – 3.6%) patients, all of whom primarily had a hematological disease. Follow-up samples of these patients were negative. *N. mikurensis* DNA was not detected in any of the blood donor samples. IgG antibodies against *B. burgdorferi* s.l. were detected with similar prevalence in immunocompromised patients and blood donors, i.e., 18/239 (7.5%, 95% CI: 4.8–11.5%) and 11/192 (5.7%, 95%: CI 3.2–10.0%).

**Conclusion:**

In this study, patients with *N. mikurensis* were not identified by clinical indication and *N. mikurensis* may therefore be underdiagnosed in Danish patients. Further investigations are needed to explore the clinical significance and implications of this infection.

## Background

Lyme borreliosis is the most well-described and prevalent tick-borne infection in Europe, but other tick-borne diseases such as neoehrlichiosis are on the rise in Europe [1–3]. Neoehrlichiosis is caused by *Neoehrlichia mikurensis* (*N. mikurensis*), an obligate intracellular bacterium of the *Anaplasmataceae* family [1, 2, 4–8]. The prevalence of *N. mikurensis* in Danish ticks is estimated to be 0.17–12.1% depending on the location [2, 5–7, 9, 10].

*N. mikurensis* seems to have low pathogenicity but can cause severe disease in immunocompromised patients [11, 12]. The known risk factors associated with severe neoehrlichiosis are splenectomy, malignant clonal B-cell disease, and B-cell depleting therapy [4, 13, 14]. The main symptom of neoehrlichiosis is prolonged fever but vascular and thromboembolic events have also been reported [11]. Other symptoms include splenomegaly, rash, cytopenia, and fatigue, which are non-specific and may be misinterpreted as another infection or even a relapse of a primary disease [14].

The use of biological therapies, for example tumor necrosis factor (TNF)-α inhibitors and monoclonal anti-CD20 antibodies, is rapidly expanding as treatments of hematological and autoimmune diseases such as B-cell lymphomas, rheumatoid arthritis, inflammatory bowel diseases, and multiple sclerosis [15, 16]. Although highly beneficial with excellent outcomes, biological therapy leaves the recipient vulnerable to infection.

Several cases of neoehrlichiosis in individuals receiving immunosuppressive therapy have been described in Europe [11, 14]. The first and only Danish case of neoehrlichiosis was published in 2020, describing a splenectomized female receiving monoclonal anti-CD20 antibodies (rituximab) as maintenance therapy for mantle cell lymphoma [17].

*N. mikurensis* is not detectable using standard routine blood culture. Further, no serological assays are available and only a few laboratories offer specific real-time polymerase chain reaction (PCR) analysis for its detection. Due to inadequate diagnostic tools and the treating physicians' unawareness of neoehrlichiosis, the infection may not be correctly diagnosed.

In this retrospective study, we investigated the prevalence of *N. mikurensis* DNA in plasma from immunocompromised patients and a group of healthy blood donors. Immunoglobulin (Ig) G antibodies against *Borrelia burgdorferi* sensu lato complex (*B. burgdorferi* s.l.) were measured as an estimate of tick exposure.

## Materials and methods

### Study design and participants

The samples used in this study were retrospectively retrieved from the Centre of Excellence for Personalized Medicine for Infectious Complications in Immune Deficiency (PERSIMUNE) Biobank and Data Warehouse, Copenhagen, Denmark. A search in the PERSIMUNE Biobank for available plasma samples was made using the following inclusion criteria: adult patients (≥ 18 years); clinical course at the Department of Hematology or Department of Rheumatology between the 1^st^ of February 2015 to the 31^st^ of December 2019; medication code for TNF-α inhibitor, recombinant monoclonal antibodies, or recombinant antineoplastic antibodies (L04AB01, L04AB02, L04AB04, L04AB05, L04AB06, L01XC02, L01XC15, L01XC17, L01XC24). In total, 239 participants during this 4-year period were considered immunocompromised either due to their hematological or rheumatological diagnosis alone or in combination with receiving immunosuppressive therapy less than a year before blood sampling. One unique plasma sample from each participant, containing 200 µL plasma, was retrieved from the biobank. Age, gender, sample date, medication initiation date, and diagnosis were the variables retrieved from the PERIMUNE data warehouse.

Plasma samples from 192 healthy blood donors donating blood in the Capital Region of Denmark (Region Hovedstaden) were retrieved from the Danish blood bank. The blood was donated between 2016 and 2019 from March to October. Age, sex, and donation date were the only available data on the blood donors.

Since whole blood is less sensitive than plasma for the detection of *N. mikurensis* by real-time PCR, plasma samples were used [18].

### DNA Purification

A total of 200 μL plasma was used for DNA purification by DNeasy Blood and Tissue Kit (Qiagen, Germany) following the manufacturer's instructions. All purified DNA was stored at − 20 °C for later analyses. A total of 500 μL plasma was used for DNA purification of the follow-up samples using the same method as mentioned above.

### Real-time PCR

A specific TaqMan probe-based real-time PCR targeting the *groEL* gene was performed to detect *N. mikurensis*. The primers and probes used have been reported elsewhere [19]. Reactions were performed in final volumes of 50 μL using 5 μL template DNA, 25 μL Platinum® Quantitative PCR SuperMix-UDG (Invitrogen, USA), 1 μM of each primer, 0.1 μM probe, 1 μM MgCl_2_, 1X ROX reference dye, and 12.7 μL water. A synthetic plasmid was used as a positive control [19], and a negative control was included in all runs. The real-time PCR conditions were as follows: initial denaturation at 95 °C for 2 min, 50 cycles of denaturation at 95 °C for 15 s, and annealing and extension at 60 °C for 1 min. A positive real-time PCR was defined as a cycle threshold (Ct) value of ≤ 36 combined with a proper sigmoid curve.

### Amplicon sequencing

Samples with detectable *N. mikurensis* DNA were further amplified by PCR with negative controls run in parallel. PCR samples were loaded on a 0.9% agarose gel stained with ethidium bromide and visualized. Amplicons were purified using QIAquick PCR Purification Kit (Qiagen, Germany) according to the manufacturer’s instructions and sequenced by Sanger sequencing by StarSEQ GmbH (Mainz, Germany) in both directions using the same primers as for the real-time PCR. The sequences were trimmed, edited, and analyzed in Geneious Prime® 2022.1.1. Forward and reverse sequences were assembled by de Novo assemble, which assembles without a reference sequence, and a consensus sequence was determined based on a threshold of 50% (bases matching at least 50% of total adjusted chromatogram qualities). Following trimming at both ends, a BLAST search was performed to confirm the identity of the sequences. A multiple alignment (MUSCLE) was performed, including *groEL* sequences from *Anaplasma phagocytophilum* (MH722254.1), *Ehrlichia ruminatium* (CR925678.1), *Candidatus* Neoehrlichia chilensis (MF805782.1), *Candidatus* Neoehrlichia lotoris (EF633745.1), and *N. mikurensis* from different geographic areas and sources (JQ359067.1, CP054597.1, KU535863.1, MG182157.1, MN701626). Genetic distances were based on the Tamura-Nei model and a phylogenetic tree was constructed according to the Neighbor-Joining method [20].

### ELISA for detection of IgG antibodies against *B. burgdorferi* s.l.

A total of 10 μL plasma was used for the detection of IgG antibodies against *B. burgdorferi* s.l. All patient and control samples were analyzed with the SERION ELISA classic Borrelia burgdorferi IgG test (Virion\Serion, Germany) following the manufacturer’s instructions and cut-off levels.

### Statistical analyses

Based on similar prevalence in cohorts presented in the literature, a specific power calculation was made. A sample size of 150 samples would allow the detection of a 2% or higher prevalence of microbial DNA with reasonable power (80%) and confidence level (95%) [21]. Fischer’s exact test was used to compare the prevalence of *N. mikurensis* DNA in the two groups. Chi-squared test for homogeneity was used to compare the seroprevalence of IgG antibodies against *B. burgdorferi* s.l. in the two groups.* p*-values < 0.05 were considered significant. All statistical analyses were performed in GraphPad Prism 9.3.1 (471).

## Results

### Study population

The study comprised 239 samples from immunocompromised patients. Baseline characteristics are summarized in Table 1. The male to female ratio was 1.4: 1 and the median age was 65 years, interquartile range (IQR) 51–73. The cohort of blood donors had a male to female ratio of 1.1: 1 and a median age of 33 (IQR 26–46). As expected, the blood donors were significantly younger (*p* < 0.0001).

**Table 1:** Characteristics of 239 patients investigated for *Neoehrlichia mikurensis*

	Number of patients
Age, median (IQR)	65 (51–73)
Male: Female	139:100
Received immunosuppressive therapy within one year prior to blood sampling, n(%)	91 (38)
**Treatment**	
Monoclonal anti-CD20 antibodies, n(%)	197 (82.4)
TNF-α inhibitors^1^, n(%)	27 (11.3)
Other^2^, n(%)	15 (6.3)
**Diagnoses**	
Lymphoma^3^, n(%)	106 (44.3)
Chronic lymphocytic leukemia, n(%)	57 (23.7)
Acute lymphocytic leukemia, n(%)	10 (4.2)
Chronic myeloid leukemia, n(%)	8 (3.3)
Acute myeloid leukemia, n(%)	8 (3.3)
Idiopathic thrombocytopenic purpura, n(%)	7 (2.9)
Myelodysplastic syndrome, n(%)	6 (2.5)
Autoimmune hemolytic anemia, n(%)	4 (1.7)
Arthritis^4^, n(%)	9 (3.7)
*Waldenström* macroglobulinemia	8 (3.3)
*Wegener’s granulomatosis,* n(%)	6 (2.5)
Systemic lupus erythematosus, n(%)	4 (1.7)
Other^5^, n(%)	7 (2.9)

Abbreviations: IQR, interquartile range; n, number; TNF, tumor necrosis factor; GVHD, graft versus host disease.

^1^Adalimumab, Golimumab, Certolizumab, Eternacept

^2^Prednisolone, Cyclophosphamide, Methotrexate, Doxorubicin, Azacitidine, Bendamustine, Obinutuzumab, Nivolumab

^3^Non-Hodgkin’s lymphoma, Hodgkin’s lymphoma, Follicular lymphoma

^4^Rheumatoid arthritis, Lupus arthritis, Spondyloarthritis

^5^Psoriasis, Graft versus host disease, Myelofibrosis

### Detection of *N. mikurensis* DNA

In total, three of the 239 (1.3%, 95% confidence interval (CI) 0.3–3.6) plasma samples from the immunocompromised patients contained detectable *N. mikurensis* DNA. None of the plasma samples from the 192 blood donors contained detectable *N. mikurensis* DNA (0%, 95% CI 0.0–1.9). The prevalence of *N. mikurensis* DNA in the two groups was not statistically different (*p* = 0.257).

### Confirmation of the detected *N. mikurensis* DNA by Sequencing

**Fig. 1:**
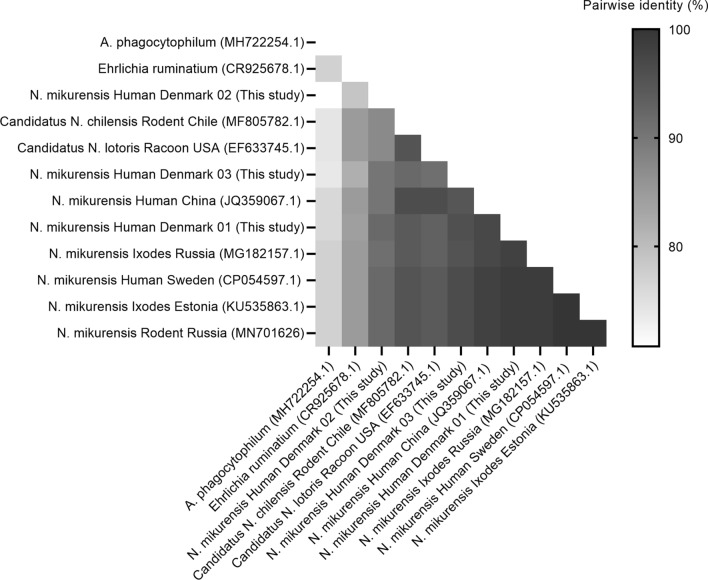
A single-color gradient pairwise nucleotide identity (%) matrix was generated from the sequences of the groEL gene from this study and selected reference sequences

**Fig. 2:**
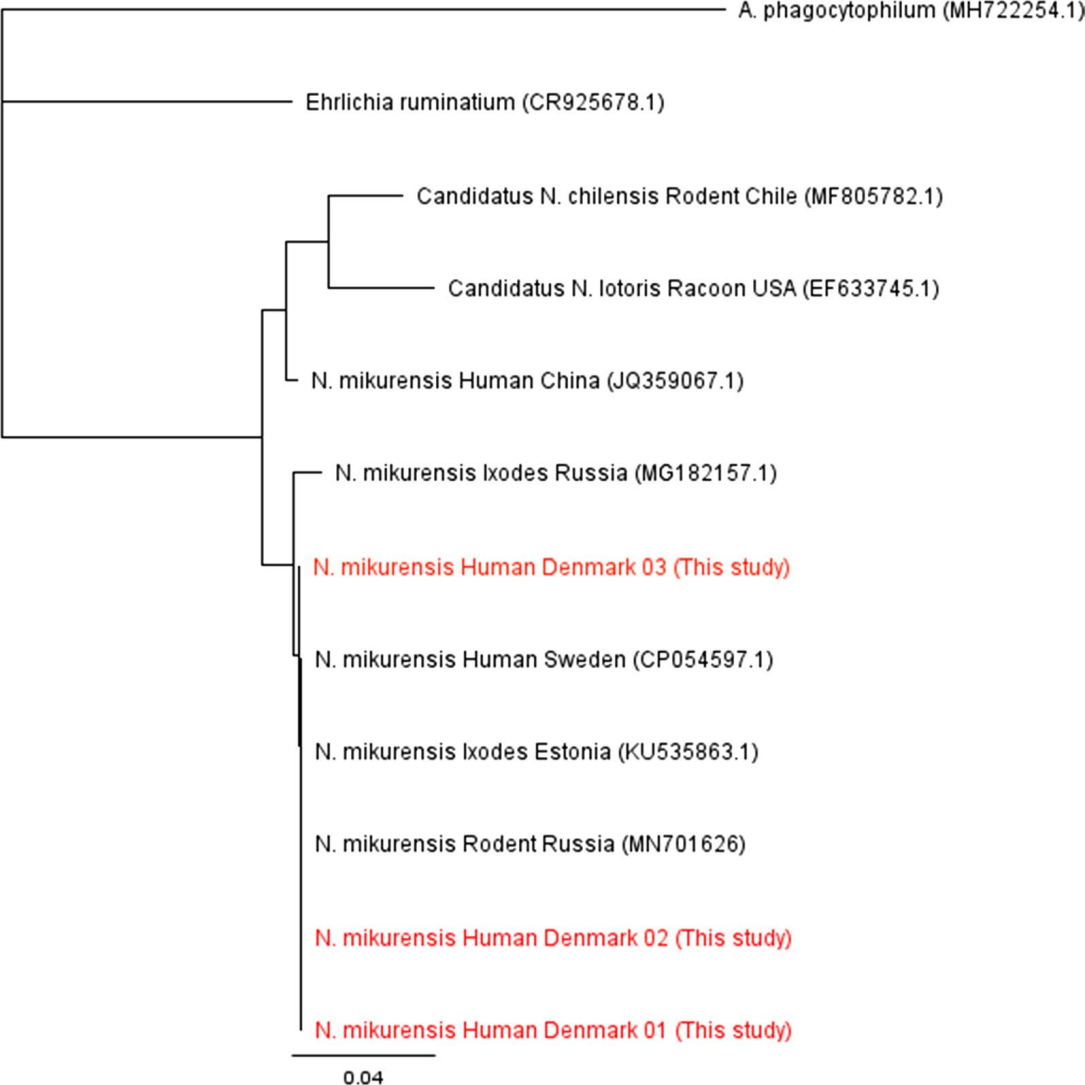
Neighbor-joining phylogenetic tree based on short sequences of the groEL gene. The relationship of the sequences from this study is compared to selected reference sequences. The sequences from this study are confirmed to be from *N. mikurensis*

A BLAST search confirmed all three sequences to be *N. mikurensis*. The percentage pairwise identity of sequences from this study and *N. mikurensis* reference sequences ranged from 90.2–99.0% (Fig. 1). Results from the Neighbor-joining tree placed the three samples from this study in the same clade as all the included *N. mikurensis* sequences, except the *N. mikurensis* from China (JQ359067.1) (Fig. 2).

### Clinical characteristics of the three cases with detectable *N. mikurensis* DNA

The three patients with detectable *N. mikurensis* DNA in a plasma sample included two females and one male (Table 2). The three patients were between 57 and 72 years at the time of blood sampling. One blood sample was collected in 2016 and two in 2019. *N. mikurensis* DNA was not detected in any of the follow-up samples collected and run by real-time PCR in 2022.

**Table 2:** Characteristics of three patients with *Neoehrlichia mikurensis* detected in a plasma sample

	**Patient 1**	**Patient 2**	**Patient 3**
Sex/age	F/57	M/72	F/66
Blood sample positive for *Neoehrlichia mikurensis *DNA	October, 2016	April, 2019	November, 2019
Diagnosis above Blood sample positive for *Neoehrlichia mikurensis* DNA	Idiopathic thrombocytopenia	B-cell prolymphocytic leukemia	Chronic lymphocytic leukemia
Immunosuppressive therapy at time of blood sampling	None^1^	Rituximab + Venetoclax initiated same day as blood sampling	Rituximab + bendamustine initiated same day as blood sampling
Time since the last dose of immunosuppressive therapy	None	Approx. 2.5 years	None
Symptoms			
Fever	No	Yes	Yes
Night sweats	No	Yes	Yes
Fatigue	No	Yes	Yes
Weight loss	No	Yes	Yes
Thromboembolic complications	No	No	No
Other symptoms	Easily bruised skin	Cough	Shortness of breath during activity, tinnitus
Hemoglobin, reference 8.3–10.5 mmol/L (male), 7.3–9.5 mmol/L (female)	9.7 mmol/L	6.0 mmol/L	5.4 mmol/L
Platelets, reference 145–390 × 10^9^/L	26 × 10^9^/L	144 × 10^9^/L	154 × 10^9^/L
C-reactive protein, reference < 10 mg/L	N/A	70 mg/L	12 mg/L
Splenomegaly (CT)	N/A	Yes	Yes
**Follow-up, May 2022**			
Status of immunosuppressive therapy	Approx. 10 months since last dose	Approx. 1.5 years since last dose	Approx. 2 years since last dose
Follow-up real-time PCR for *Neoehrlichia mikurensis* DNA	Negative	Negative	Negative
Antibiotics registered from blood sample to follow-up sample	None	Amoxicillin/clavulanic acid Ciprofloxacin Pivmecillinam Trimethoprim Nitrofurantoin Piperazilin/ tazobactam Azithromycin	Piperazilin/ tazobactam Sulfamethoxazole-trimethoprim

CT, computerized tomography; PCR, polymerase chain reaction; N/A, not applicable

^1^The patient did not start immunosuppressive therapy (Rituximab) until after blood sampling, the only immunosuppressive therapy prior to blood sampling was budesonide

^2^Only antibiotics prescribed from the hospital

### Exposure to ticks by analyses of IgG antibodies against *B. burgdorferi* s.l.

In total, 18/239, (7.5%, 95% CI 4.8–11.6) of the plasma samples from the immunocompromised patients and 11/192 (5.7%, 95% CI 3.2–10.0) of the blood donors had detectable IgG antibodies against *B. burgdorferi* s.l. None of the three patients with detectable *N. mikurensis* DNA had detectable IgG antibodies against *B. burgdorferi* s.l. The seroprevalence of *Borrelia*-specific IgG antibodies was not significantly different (*p* = 0.563) in the immunocompromised patients compared with the blood donors.

## Discussion

In this retrospective study, *N. mikurensis* DNA was detected in 1.3% (3/239) of the immunocompromised patients. All three patients had a hematological diagnosis. Follow-up samples were all negative and none of the 192 blood donor samples had detectable *N. mikurensis* DNA. Thus, testing for *N. mikurensis* in immunocompromised patients should be considered in a Danish setting. Future awareness of *N. mikurensis* among physicians is important to diagnose neoehrlichiosis and avoid diagnostic delay in this group of patients.

Denmark, like most of Europe and Scandinavia, is a tick endemic area, with an estimated 73.5% of the population living within 5 km of areas with tick nymphs [22]. *B. burgdorferi* s.l. is the most prevalent human pathogen in Danish ticks [7]. Based on the seroprevalence of *Borrelia*-specific IgG antibodies, tick exposure was not significantly different between the patient cohort and the blood donors (7.5% vs. 5.7%). The seroprevalence is in accordance with another recent Danish study reporting a seropositive rate of 7% in blood donors [23].

*N. mikurensis* has been detected in ticks from more than 18 countries in Europe with a prevalence varying from 0.3% in Poland to 25.5% in Norway [1, 24–27]. The prevalence of *N. mikurensis* in blood from immunocompromised patients and healthy blood donors found in this study is comparable to or slightly lower compared to our neighboring Scandinavian countries [12, 28]. This agrees with a lower prevalence of *N. mikurensis* in ticks collected in Denmark compared to Norway [7]. A study from southern Norway found the prevalence of *N. mikurensis* in a cohort of immunocompromised patients living in a tick endemic area to be 7.4% (12/163), collected in 2018 (September–December) and 2019 (March–May), and 1.2% (1/85) in a cohort of immunocompetent controls, collected in 2013/2014 [12]. A recent study from southeastern Sweden found a prevalence of 0.7% among 1006 blood donors, collected in 2019 (June–August) and 2021 (February–November) [28]. The samples from our cohort of immunocompromised patients were collected all year round, and the blood donor samples were collected from March to October. Hence, our findings may have been influenced by sampling outside “tick-season”. However, *N. mikurensis* has been found to persist in the bloodstream for longer periods among immunocompromised as well as in immunocompetent individuals [12, 28], although re-infection is also possible. Considerably higher prevalence was found among symptomatic individuals, with recent tick-bite exposure in Norway (10%) and Sweden (1.9%) [18, 19]. This suggests that the symptomatology of the patients under investigation and their immunological health may have a greater influence on the reported prevalence than the time of year the blood sample was collected.

The only published case of human neoehrlichiosis in Denmark describes a patient treated with rituximab, who presented with fever and a persisting rash despite antibiotic treatment [17]. Since then, three cases of neoehrlichiosis have been diagnosed at Copenhagen University Hospital, Rigshospitalet, and all three were receiving rituximab for a primary disease [29, 30]. The use of biological therapy in a variety of medical specialties is rising [15, 31, 32]. Considering the increased use of immunosuppressive therapy, the findings in the present study and the increasing number of published clinical cases around Europe, the prevalence of *N. mikurensis* might be higher than expected. More countries are being added to the list of published cases of neoehrlichiosis, most recently France [33], Slovenia [34], and Germany [35].

Two of three patients had symptoms attributed to neoehrlichiosis around the time *N. mikurensis* DNA was detected in blood, although given the retrospective nature of the study it is not definitive and could also be attributed to the hematologic malignancy or another undetected infection. No doxycycline/rifampicin treatment was documented in the three cases, but treatment could have been prescribed in a primary healthcare setting. Currently, no method for antibiotic susceptibility testing exists for *N. mikurensis*, and based on published cases other broad-spectrum antibiotics such as piperacillin/tazobactam, meropenem, ciprofloxacin, levofloxacin, clindamycin, cefotaxime, ceftazidime, and gentamycin are largely ineffective [4]. Since patient 2 and 3 did not receive immunosuppressive therapy for 1.5 and 2.5 years prior to the follow-up samples (Table 2), the patients may have acquired immunocompetence by the time of the follow-up, which would account for the negative follow-up samples. Patient 1 was included as a patient with assumed impaired immune system, but according to the medical record, she did not receive immunosuppressive therapy prior to the first blood sample. It is, however, not surprising as asymptomatic *N. mikurensis* infection has been described in both immunocompromised and immunocompetent individuals [12, 18]. Interestingly, no thromboembolic or vascular events were reported by any of the three patients, although especially thrombophlebitis and deep vein thrombosis have been associated with *N. mikurensis* infection in up to 63% of immunocompromised patients and 50% in immunocompetent individuals [11].

Our phylogenetic analyses of *N. mikurensis* DNA from Danish patients suggest a close relationship with isolates found in Sweden, Estonia, and Russia (Fig. 2), supporting that birds most likely disperse *N. mikurensis* infected ticks over large geographical areas as a part of their natural migration patterns [36].

Given the results from the current study and recent clinical cases from all over Europe, testing for *N. mikurensis* in immunocompromised patients as part of the standard investigation seems reasonable in a Danish setting as well [33].

### Limitations and perspectives

The study is limited by its retrospective design, as it is not possible to ascertain that the clinical manifestations around the time of the initial blood sample in our three cases were caused by neoehrlichiosis or by another condition. No consecutive blood samples from patients with detectable *N*. *mikurensis* DNA were available in the biobank, which would have allowed us to follow the *N. mikurensis* bacteremia. Uncertainty remains about the time of administration of immunosuppressive therapy, some have likely received immunosuppressive therapy for the first time after the blood sampling, whereas other participants have received other types of immunosuppressives before blood sampling, and we were limited by only having data on specific immunosuppressive therapy. There was no statistically significant difference in outcome parameters between immunocompromised and blood donors. However, the analysis was underpowered, based on previous studies, we designed the study to detect a difference of 2%. A power calculation based on our newly found 1.3% among immunocompromised patients, would allow us to detect a significant difference between groups with a needed sample size of 600 in each group [21]. However, the sample size was not available for this study.

## Conclusion

*Neoehrlichia mikurensis* DNA was detected in 1.3% of a cohort of immunocompromised patients indicating that neoehrlichiosis is likely underdiagnosed in Danish patients. Additionally, it emphasizes the likelihood of *N. mikurensis* being a risk in Danish patients. Therefore, prospective studies are needed to explore the clinical significance and implications in this group of patients. Screening patients receiving B-cell depleting therapy and presenting with fever for *N. mikurensis* could be relevant.

## Data Availability

All data generated or analysed during this study are included in this published article [and its additional information files].

## Abbreviations

*N. mikurensis*: *Neoehrlichia mikurensis*
TNF-α: tumor necrosis factor-α
PCR: Polymerase Chain Reaction
Ig: Immunoglobulin
*B. burgdorferi* s.l.: *Borrelia burgdorferi* sensu lato complex
PERSIMUNE: Personalized Medicine for Infectious Complications in Immune Deficiency
